# Formulation and evaluation of atovaquone-loaded macrophage-derived exosomes against *Toxoplasma gondii*: *in vitro* and *in vivo* assessment

**DOI:** 10.1128/spectrum.03080-23

**Published:** 2023-11-28

**Authors:** Fatemeh Goudarzi, Vahid Jajarmi, Saeedeh Shojaee, Mehdi Mohebali, Hossein Keshavarz

**Affiliations:** 1 Department of Medical Parasitology and Mycology, School of Public Health, Tehran University of Medical Sciences, Tehran, Iran; 2 Department of Medical Biotechnology, School of Advanced Technologies in Medicine, Shahid Beheshti University of Medical Sciences, Tehran, Iran; 3 Center for Research of Endemic Parasites of Iran (CREPI), Tehran University of Medical Sciences, Tehran, Iran; University of California, Riverside, Riverside, California, USA

**Keywords:** drug delivery, exosome, atovaquone, toxoplasmosis, *in vitro*, *in vivo*

## Abstract

This study aimed to prepare and assess atovaquone (ATQ)-loaded exosomes (EXO-ATQ) against *Toxoplasma gondii* to improve their therapeutic activities on acute and chronic phases of murine toxoplasmosis. Exosomes were isolated from a mouse macrophage cell line (J774A.1). The isolated exosomes were loaded with ATQ (EXO-ATQ), applying the co-incubation method. *In vitro* efficacy of EXO-ATQ against *T. gondii* infection was assessed in Vero cell culture. An *in vivo* experiment was carried out in BALB/c mice infected with RH (acute strain) and Tehran (chronic strain) of *T. gondii,* followed by treatment with EXO-ATQ. Their survival time and parasite load were compared with that in the suspension of ATQ (S-ATQ) as a positive control group. The *in vitro* study showed that exosomes improved the efficacy of ATQ, and EXO-ATQ significantly reduced intracellular proliferation of *T. gondii* tachyzoites compared to S-ATQ (*P*  ≤  0.05). In acute experimental toxoplasmosis, the mice treated with EXO-ATQ showed a significant reduction in tachyzoites count of the peritoneal cavity and a longer survival time (*P*  ≤  0.05). Furthermore, EXO-ATQ administration significantly decreased the mean number (97.3% cyst reduction) and the average size of the brain cysts of chronically infected mice with *T. gondii*, Tehran strain (*P*  ≤  0.05). Besides, the decrease in cyst numbers was confirmed by the down-regulation of *BAG1* using a real-time PCR assay. Our results implied that exosomes have the potential to be used as an efficient drug delivery system, and the loading of ATQ into the exosomes ameliorates the effect of ATQ against *T. gondii*. This study suggests a new strategy for improving the effectiveness of ATQ against acute and chronic phases of *T. gondii*.

**IMPORTANCE:**

This study is the first of its kind that suggests exosomes as a nano-carrier loaded with atovaquone (ATQ), which could be considered as a new strategy for improving the effectiveness of ATQ against acute and chronic phases of *Toxoplasma gondii*.

## INTRODUCTION

*Toxoplasma gondii* is an obligate intracellular parasite with a global distribution that virtually infects all warm-blooded animals, including humans (1, 2). *T. gondii* infection is generally asymptomatic in immunocompetent individuals (3). However, in immunocompromised persons, such as HIV-positive and cancer patients, as well as transplant recipients, it is a life-threatening disease primarily due to the reactivation of *T. gondii* cysts in the brain (4). The most widely used treatment for the management of toxoplasmosis is the combination of pyrimethamine and sulfadiazine (5). Despite the rapid and significant efficiencies of this combination, several side effects such as hematologic toxicity, allergy, bone marrow suppression, and folic acid deficiency have been reported, which are considered as the main limitation of using these drugs (6). Furthermore, this combination is not effective against tissue cysts (7). Despite significant advances in pharmacological research, treatment of the chronic phase of this disease is still challenging due to poor drug delivery across the blood-brain barrier (BBB). Furthermore, there are no effective and efficient vaccines for widespread use in humans. Therefore, developing drugs that affect all stages of the parasite, especially the cystic form, is critical. Atovaquone (ATQ), a hydroxynaphthoquinone, is the only successful treatment against tissue cysts during chronic infection with the mechanism of blocking the respiratory chain of the parasite (8, 9). ATQ presents limited efficacy due to high hydrophobicity and low bioavailability, but new formulations and drug delivery systems may improve the activity of ATQ (10, 11). Extracellular vesicles (EVs) are the carrier of choice that can be used via intravenous (IV) route (12) and offer several advantages over synthetic drug delivery systems, including low toxicity, immune compatibility, and robust targeting (13). EVs are small membrane-bound vesicles with different size ranges (40–1,000 nm) produced by most mammalian cells under physiological and pathological conditions (14). Exosomes or small EVs (40–150 nm) are derived by the invagination of endosomal membranes (i.e., multivesicular bodies) and their fusion with the plasma membrane (15). Compared to nanoparticles (NPs) and liposomes, exosomes offer more biocompatibility, low immunogenicity, the capacity to cross biological barriers easily, and non-toxic aggregation. According to this criterion, exosomes are suitable carriers for drug delivery (16). Exosomes from varied cell types have been used as delivery vehicles for a variety of therapeutic agents, including proteins (17), both macromolecules (DNA and RNA) (18), small molecules (19), doxorubicin (20), curcumin (21), paclitaxel (22), ATQ (23), sorafenib (24), and porphyrins (25). Most of these studies have focused on cancer treatment, but EVs could be applied to treat other pathologies such as Alzheimer’s (26) and Parkinson’s disease (27), malaria (23), cerebral occlusion (28), and ischemic kidney injury (29). Considering that no study has been conducted on the use of exosome for the treatment of toxoplasmosis, in this study, for the first time, exosomes derived from a mouse macrophage cell line (J774A.1) were used as a nano-carrier loaded with ATQ (EXO-ATQ), against acute and chronic phases of *T. gondii in vitro* and *in vivo* compared with the suspension form of ATQ (S-ATQ).

## MATERIALS AND METHODS

### Materials

ATQ was purchased from Hubei Vanz Pharm, China (Ca No. 95233–18-4). The exosome isolation kit was provided by Cib Biotech Co. (Tehran, Iran). 3-(4,5-Dimethylthiazol-2-yl)−2,5-diphenyltetrazolium bromide (MTT), RPMI-1640, fetal bovine serum (FBS), and penicillin/streptomycin were obtained from Sigma-Aldrich, USA. The bicinchoninic acid (BCA) protein quantification kit was purchased from DNA Biotech Co (Tehran, Iran). Dimethyl sulfoxide (DMSO) and ethanol were supplied by Merck, Germany.

### Parasite strains and cell lines

The virulent RH strain (Type Ι) and avirulent Tehran strain (Type ΙΙ) (30) of *T. gondii* were maintained in BALB/c mice using serial subculture in the Department of Medical Parasitology and Mycology, School of Public Health, Tehran University of Medical Sciences, Tehran, Iran.

The mouse macrophage J774A.1 cell line and Vero cell line were obtained from the National Cell Bank of Iran (NCBI, Pasteur Institute of Iran, Tehran, Iran) and cultured in RPMI 1640 medium supplemented with 10% heat-inactivated FBS and 1% penicillin-streptomycin (10,000 U/mL). Cell lines were incubated at 37°C in a humidified incubator containing 5% CO_2_. After that, the cells were passaged by trypsinization when 70%–80% confluency was reached at seeding densities of 5 × 10^5^ cells/mL.

### Mice

Six- to eight-week-old female BALB/c mice were provided by the Razi Institute (Karaj, Iran). The animals were raised, handled, and treated according to ethical guidelines of animal treatment and handling of the Institutional Ethical Committee and Research Advisory Committee of Tehran University of Medical Sciences (IR.TUMS.SPH.REC.1400.004). The mice were maintained in cages held at room temperature (22°C–24°C) in a ventilated room with unrestricted access to water and food.

### Cell adaptation and isolation of exosomes

Due to the presence of exosomes in FBS (31), to avoid their interference with macrophage exosomes, the mouse macrophage J774A.1 cell line was adapted to the FBS-free medium through sequential adaptation. The adaptation process started with cells in the exponential growth phase at 3 × 10^5^ cells/mL cell density and cell viability of >90%. The percentage of FBS gradually decreased from 10% to 0% over 10 days in the culture medium. The adapted cells were cultured in T75 flasks when the cells were stabled in a serum-free medium without considerable morphological changes. The flasks were kept at 37°C in a humidified incubator containing 5% CO_2_ for 48 h, and a conditioned medium was applied for exosome isolation. Exosomes were isolated from a conditioned medium using Exocib kit (Cibzist, Tehran, Iran) according to the manufacturer’s protocols. Briefly, particles and cell debris were removed by centrifugation at 300 × *g* for 10 min, followed by filtration through a 0.22 µm membrane to remove large vesicles. In the next step, reagent A was added to the filtered medium at a ratio of 1:5 (reagent A 1: sample 5). The mixed sample was vortexed thoroughly for 5 min and incubated overnight at 4°C. Subsequently, the sample was vortexed for an additional 1 min and centrifuged at 3000 × *g* for 40 min at 4°C. Finally, the supernatant was discarded entirely, and exosomes were resuspended in reagent B and stored at −80°C for the following experiments.

### Characterization of isolated exosomes

The protein concentration of isolated exosomes was measured using the BCA protein quantification kit (DNA Biotech, Tehran, Iran). To determine the size and zeta potential of exosomes, we employed the Nano Zetasizer (Cordouan Technologies, France). For this purpose, 50 µL of exosomes was diluted in 950 µL of phosphate-buffered saline (PBS). Prior to the measurements, the Nano Zetasizer was blanked using PBS as a reference. Subsequently, the size distribution and zeta potential of the exosomes were measured at 25°C, using a refractive index of 1.38 and an absorption value of 0.01. Transmission electron microscopy (TEM) was used for the morphological assessment of the isolated exosomes. Briefly, the isolated exosomes were fixed for 30 min with 2% paraformaldehyde at room temperature and then loaded on TEM grids treated with UV light to resolve static electricity. For scanning electron microscopy (SEM), the exosomes were fixed with 2.5% glutaraldehyde for 15 min. After that, the exosomes were washed with PBS and then serially dehydrated with an ascending sequence of ethanol (30%, 50%, 60%, 80%, and 98%). The samples were left to dry at room temperature and then analyzed by SEM.

Bead-based flow cytometry assay was used to investigate the presence of vesicle markers in exosomes, including CD9 and CD63. Briefly, the exosomes were coupled to 4 μm aldehyde/sulfate latex beads (Invitrogen, A37304) to invert them to cell scale and then incubated with anti-CD9 and anti-CD63 antibodies for flow cytometry analysis. We used 40 µg of exosomes with 10 µg well-shaken beads, and after overnight incubation, the washing process was done with PBS and glycine. Then, the exosome-bead complex was incubated with CD9 and CD63 antibodies and subjected to flow cytometry (BD FACS Caliber). Data were analyzed with FlowJo 7-6-1 software.

### Loading of ATQ by incubation

In order to load the exosomes with ATQ, naive exosomes were diluted in PBS and then ATQ solution was added and incubated for 12 h at room temperature under stirring. The theoretical concentration ratio of ATQ to exosome proteins (determined by the BCA assay) was kept at 1:2. After passing through a 100 KDa amicon filter to eliminate free ATQ, the loaded ATQ was quantified spectrophotometrically. From the free ATQ available in the supernatant, the entrapped ATQ was calculated and expressed as loading efficiency.

Loading efficiency = [(amount of ATQ loaded in exosomes)/(amount of ATQ initially added)] × 100

After preparing the loaded ATQ, Nano Zetasizer was applied to distinguish the zeta potential and size distribution of EXO-ATQ.

### Cytotoxicity assay

The cytotoxic activity of S-ATQ, J774A.1 EXO, and EXO-ATQ was evaluated by the MTT assay. Briefly, Vero cells were seeded at a density of 3 × 10^3^ cells/well in a 96-well plate. After overnight seeding, all culture medium supernatants were removed from the wells, and the cells were exposed to different concentrations of S-ATQ, J774A.1 EXO, and EXO-ATQ (62.5, 125, 250, 500, and 1,000 µg/mL) for 48 h. Then, 20 µL of MTT solution was added to each well, followed by continuous incubation for 4 h. After incubation, the culture medium was carefully discarded, and 100 µL of DMSO was added to each well to dissolve the formazan crystals. Triplicate results were read by an ELISA reader at 570 nm, and cell viability was calculated based on the equation: (absorbance of the treated sample/absorbance of the control cells) × 100.

### Intracellular proliferation assay

Vero cells (2  ×  10^5^/well) were cultured in RPMI medium in 8-well cell culture microplates (SPL, South Korea) at 37°C with 5% CO_2_ for 24  h. Adherent cells were infected with tachyzoites of *T. gondii*, RH strain, at a ratio of 1:3 parasite/cell. After 3  h, non-adherent cells and free parasites were removed by washing with RPMI. Then, concentrations of 15, 30, 60, 120, and 240  µg/mL of each compound (S-ATQ, J774A.1 EXO, and EXO-ATQ) were added to microplates, and one well received no treatments as control. Each experiment was carried out in triplicate. After 48 h, microplates were rinsed, fixed, and stained with Giemsa dye for 20 min. The cells were analyzed with a light microscope to determine the *T. gondii* infection rate (number of infected cells per 100 examined cells) and parasite intracellular replication (mean number of the intracellular parasite in 100 infected cells).

### *In vivo* assay

#### Initial assessment of IV administration of EXO-ATQ in mice

Overall, eight female BALB/c mice in two groups (group 1: EXO-ATQ and group 2: PBS) were used in this part of the study. Four mice were injected with EXO-ATQ (10 mg/kg/every 3 days) at regular 72 h intervals for 3 weeks, and four mice were injected with PBS as a control group. The injection was done by lateral tail vein using a 30-gauge needle under aseptic conditions. Mice were weighed before and at the end of the study. The behavior and physical appearance of the control and treated groups were monitored and recorded daily. On the last day, the animals were euthanized, and their blood was taken intracardially to determine the aspartate transaminase (AST), alanine aminotransferase (ALT), and alkaline phosphatase (ALP) levels. Subsequently, livers and spleens were weighed and fixed with 10% formalin, embedded in paraffin, and cut into 5-μm-thick sections for histological analysis.

#### Treatment of acute toxoplasmosis

To evaluate the effects of EXO-ATQ in comparison with S-ATQ on the acute phase, sixty 6–8-week-old female BALB/c mice weighing 20–25 g were randomly divided into six groups (*n* = 10) according to the following experimental setup: One group did not inoculate with any tachyzoite as a healthy control, and mice in other five groups were inoculated intraperitoneally with *T. gondii* tachyzoites (RH strain, 1 × 10^4^ tachyzoites per mouse); 4 h later, all inoculated groups were treated as follows:

Group 1, IV injection of the PBS as a control group; Group 2, IV injection of the S-ATQ (10  mg/kg); Group 3, IV injection of the EXO-ATQ (10  mg/kg); Group 4, IV injection of the J774A.1 EXO; and Group 5, IV injection of the DMSO as a solvent for ATQ. The treatment lasted for 9 days, and the compounds were administrated intravenously every 3 days. Survival was checked daily, and the mortality rate was documented for each group until all mice had died. To assess the parasite burden, the peritoneal cavity of five mice from each group was aspirated on day 4 and day 6 post-infection. Parasite load was evaluated by counting the number of tachyzoites using optical microscopy at 400× magnification. To assess the virulence potential of post-treated tachyzoites, a parasite suspension of each group (1 × 10^4^ tachyzoites per mouse) was inoculated to new groups of mice (*n* =  5), and the survival time of each group was recorded.

#### Treatment of chronic toxoplasmosis

Chronic toxoplasmosis was studied using 60 female BALB/c mice aged 6–8 weeks. To establish chronic infection, mice were infected with the intraperitoneal injection of 10 tissue cysts of *T. gondii*, Tehran strain. Three weeks post-infection, the mice were divided into six groups as detailed in the acute phase study, and treatment was carried out for 18 days. On the last day, mice were sacrificed, and their brains were harvested and divided into two hemispheres under a laminar-flow tissue culture hood. The right hemisphere was submerged in liquid nitrogen and stored at −80°C for *bradyzoite surface antigen 1* (*BAG1*) gene expression, and the left hemisphere was used for calculating the tissue cysts. Impression smears of the mice brain tissues were prepared, the number of cysts was counted, and the size of cysts was calculated using light microscopy at 100× and 400× magnifications, respectively. The following equation was used to estimate the % reductions (%R) of cysts: {%R = 100 × [(C − E)/C]; where C: control group and E: experimental groups of mice}.

A SYBR green-based real-time PCR was used to assess the BAG1 gene expression. Total RNA from the brain tissues was extracted using the Total RNA Extraction Mini Kit (Favorgen, Taiwan) according to the manufacturer’s protocols and stored at −80°C until use. cDNA was synthesized using the cDNA Synthesis Kit (YTA, Iran). The expression levels of the *BAG1* gene were determined using the SYBR Green Master Mix Kit (Ampliqon, Odense, Denmark) on a StepOne Real-time PCR System (Applied Biosystems, USA). The gene-specific primer sets for *BAG1* as the target gene and *glyceraldehyde 3-phosphate dehydrogenase* (*GAPDH*) as the housekeeping gene have been used for real-time PCR (32). The primer sequences for *BAG1* included forward (5′-AGTCGACAACGGAGCCATCGTTATC-3′) and reverse (5′-ACCTTGATCGTGACACGTAGAACGA-3′) and for *GAPDH* included forward (5′-GAGAAACCTGCCAAGTATG-3′) and reverse (5′-TGTAGCCGTATTCATTGTC-3′). During real-time PCR, the conditions of temperature were an early denaturation at 95°C for 15 min followed by 40 cycles of 95°C for 15 s, 60°C for 15 s, and 72°C for 15 s, with the final elongation step at 72°C for 5 min. All tests were performed in triplicate to guarantee replicability, and the data were analyzed by calculating 2^−ΔΔCT^.

To evaluate the virulence potential of post-treated parasites, new mice groups (each of five) were inoculated intraperitoneally by the suspension of tissue cysts of the first groups (10 tissue cysts per mouse). After 6 weeks, mice were sacrificed, and cysts were counted in brain tissues.

### Statistical analysis

Data are represented as the mean ± SD. Differences between groups were determined using independent samples *t* test or analysis of variance (ANOVA). The Kaplan–Meier method was used to determine the mice survival rate. Results were analyzed using SPSS (version 26; IBM Corp., Armonk, NY, USA) and GraphPad Prism 9.0 software. The statistical significance threshold was set as *P* < 0.05.

## RESULTS

### Characterization of J774A.1 EXO

**Fig 1:**
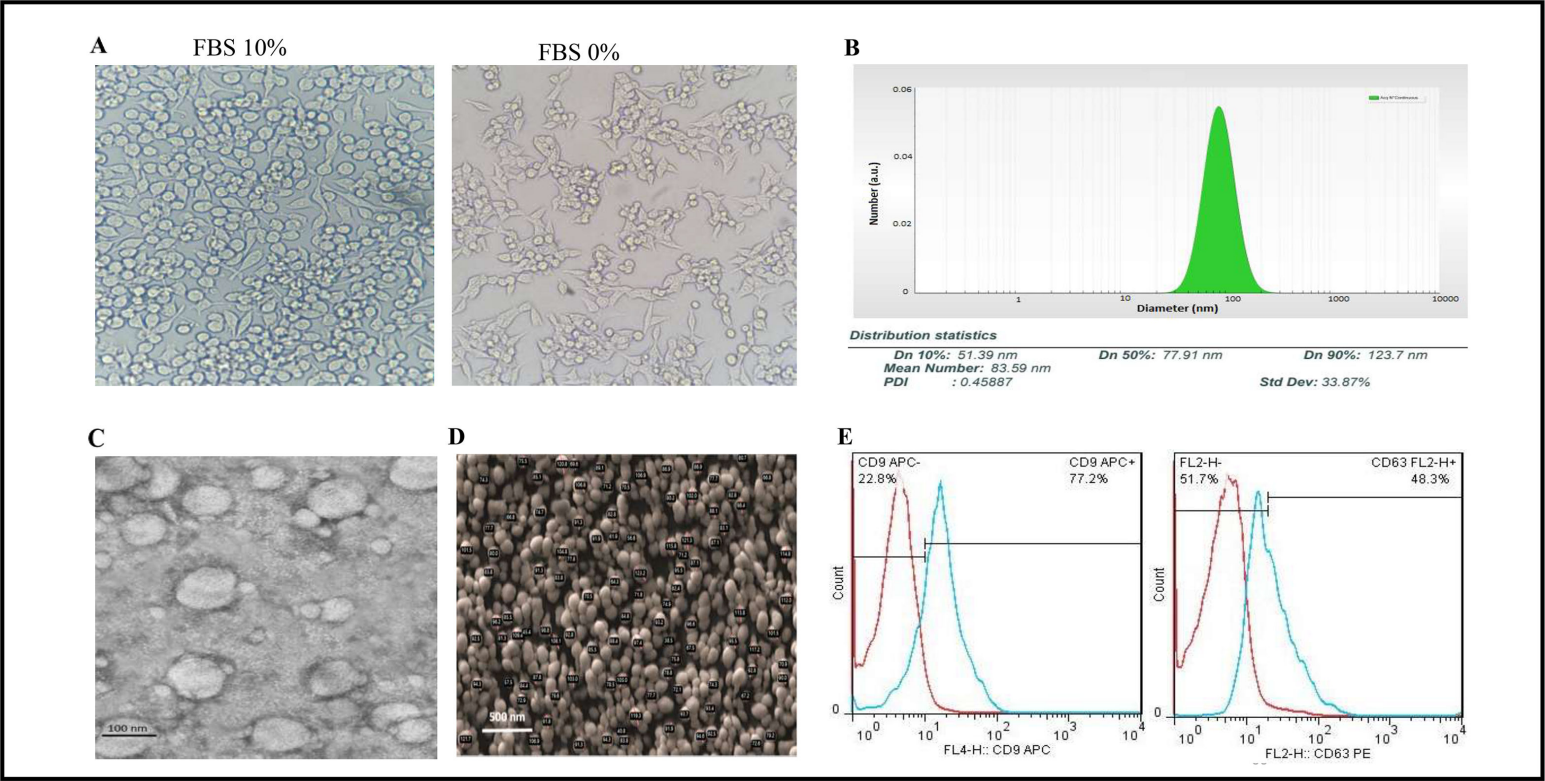
Characterization of J774A.1 EXO. (**A**) Morphology of J774A.1 cells in two different conditions, FBS 10% and FBS 0%, under an inverted microscope. No significant morphological changes were detected. (**B**) Size distribution of J774A.1 EXO using dynamic light scattering (DLS) showed an average diameter of 84 nm. (**C**) TEM image of J774A.1 EXO showed the typical structure of exosomes. (**D**) SEM image of J774A.1 EXO showed spherical particles with diameters ranging between 38 and 128 nm. (**E**) Flow cytometry analysis revealed that isolated exosomes were positive for CD9 and CD63 exosomal markers.

The J774A.1 cells were adapted to FBS-free medium through sequential adaptation (Fig. 1A). According to the BCA method, the average yield of exosomes isolated from 100 mL of the J774A.1 cell culture supernatant was 2,024 µg. Based on the Nano Zetasizer results (Fig. 1B), the average size and zeta potential of J774A.1 EXO were determined to be 84 nm and −13 ± 1 mV, respectively. Moreover, TEM (Fig. 1C) and SEM (Fig. 1D) analyses revealed the cup shape of exosomes and the average size to be between 38 and 128 nm. Analysis of phenotypic markers showed that isolated exosomes were positive for CD9 and CD63 (Fig. 1E).

### ATQ assessment

**Fig 2:**
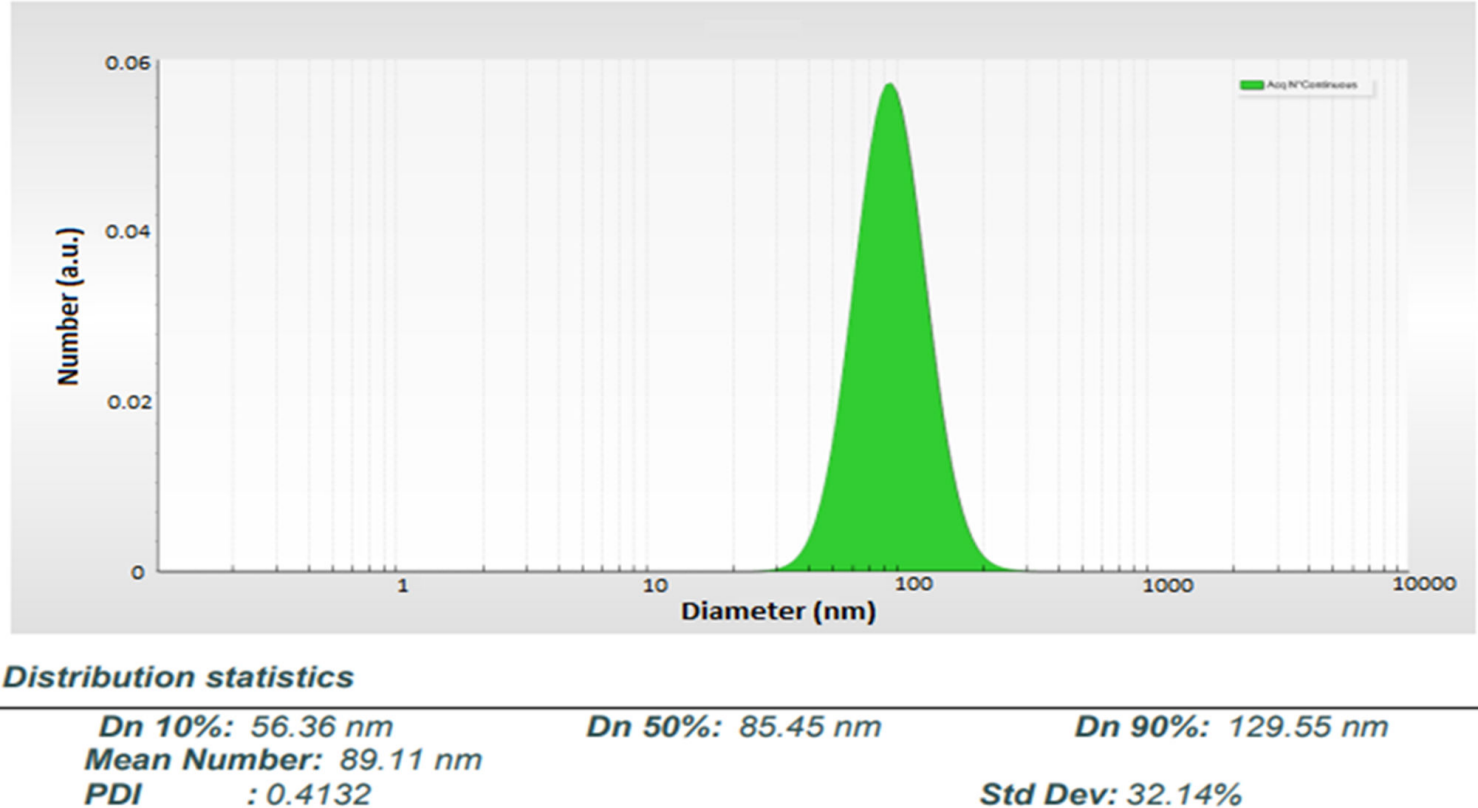
Size distribution of EXO-ATQ using dynamic light scattering. The peak diameter was about 89 nm.

The isolated exosomes were loaded with ATQ, applying the co-incubation method. The average loading efficiency of ATQ into the exosomes was 57% ± 6%. After the preparation of loaded ATQ, the size and zeta potential of EXO-ATQ were assessed using Nano Zetasizer. According to the result, the average diameters and zeta potential of EXO-ATQ were 89 nm (Fig. 2) and −14 ± 1 mV, respectively, similar to naive exosomes.

### Cytotoxicity assay

**Fig 3:**
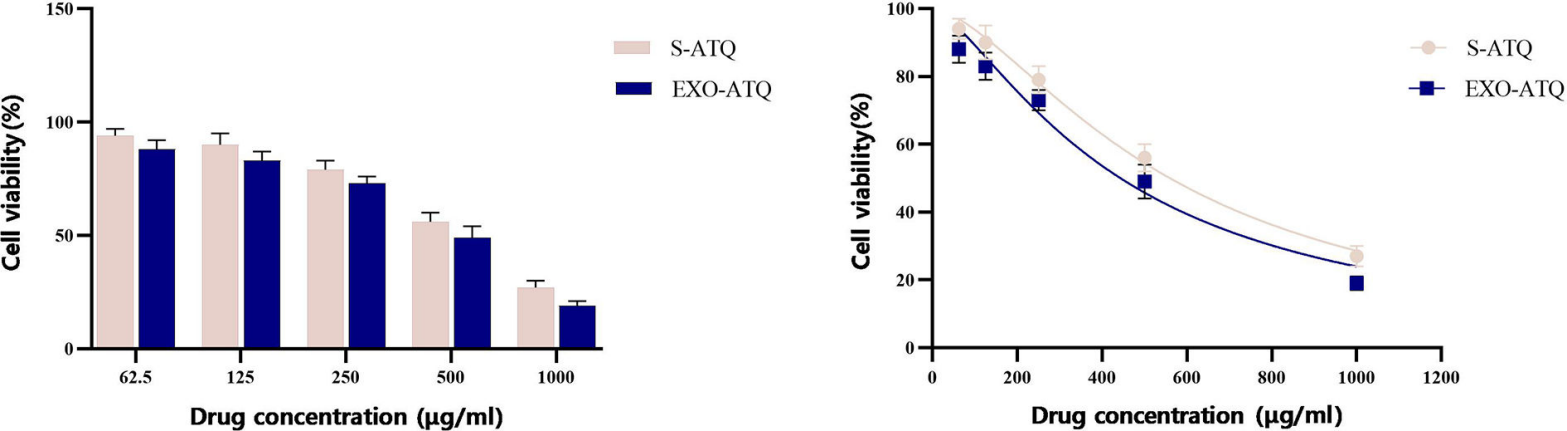
Cell viability assessment by MTT assays. Vero cells were treated with ATQ at different concentrations with or without exosomes for 48 h. S-ATQ and EXO-ATQ considerably reduced cell viability in a concentration-dependent manner with no significant differences (*P* > 0.05). Independent samples *t* test was used to make comparisons between groups.

The cytotoxicity of S-ATQ, J774A.1 EXO, and EXO-ATQ on Vero cells was investigated by the MTT assay, and the half-maximal cytotoxic concentration (CC50) for each compound was calculated using nonlinear regression analysis of GraphPad Prism software (version 9.0). The results indicated that J774A.1 EXO had no significant effects on the viability of Vero cells. However, administration of S-ATQ and EXO-ATQ caused a significant concentration-dependent decrease in cell viability compared to control (untreated cells). At the time of 48 h, CC50 values for S-ATQ and EXO-ATQ were 560 and 443 µg/mL, respectively, and no significant differences were found between the cytotoxicity of S-ATQ and EXO-ATQ (Fig. 3).

### Intracellular proliferation of *T. gondii* tachyzoites

**Fig 4:**
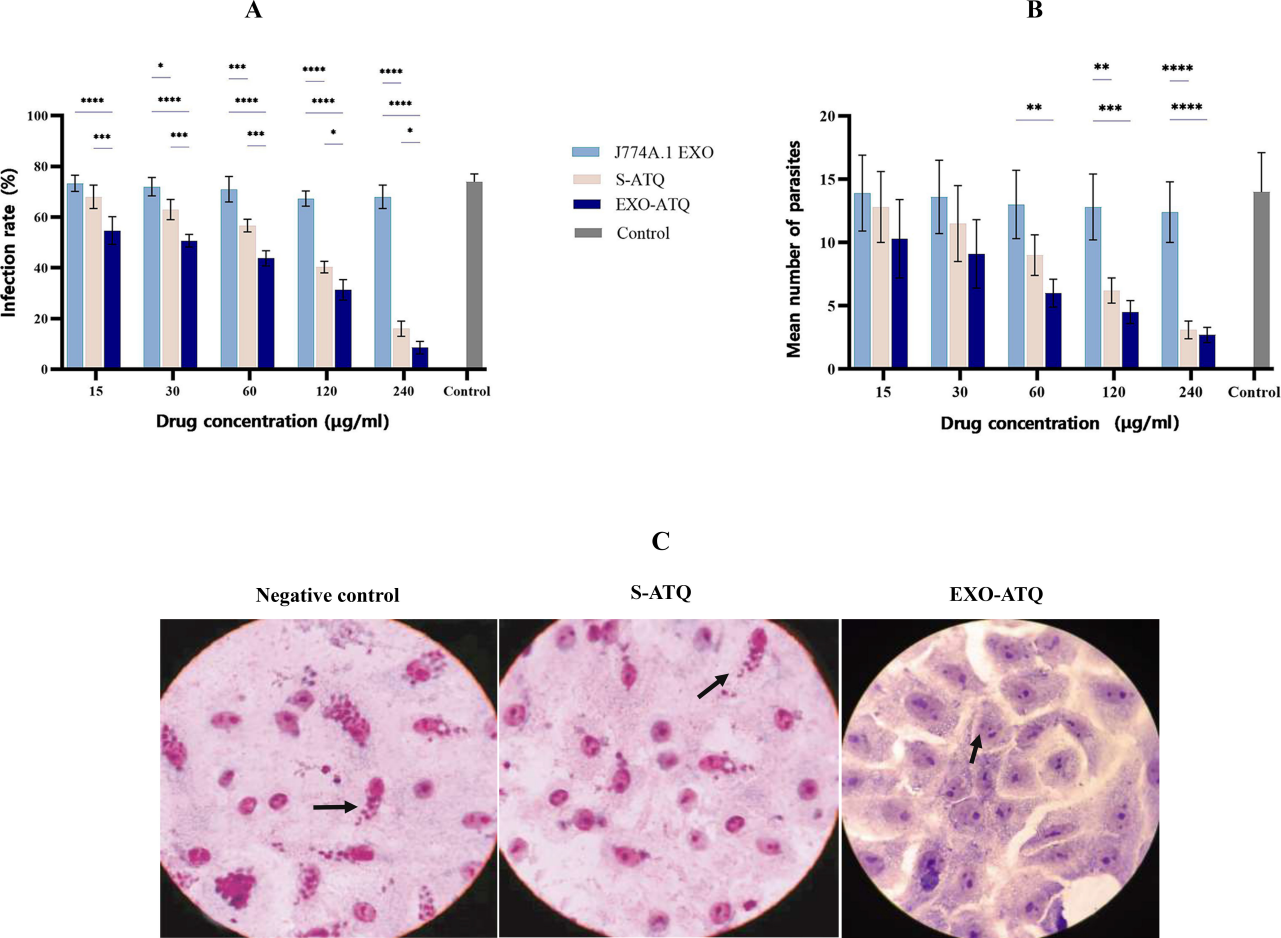
(A) Infection rate and (**B**) the mean number of intracellular tachyzoites in the infected cells at different concentrations of compounds. EXO-ATQ significantly reduced the infection rate of *T. gondii* tachyzoites. The data represent three independent experiments and are presented as mean ± SD. One-way ANOVA was used to make comparisons between groups. *P < 0.05, **P < 0.01, ***P < 0.001, and ****P < 0.0001. (**C**) Microscopic images of tachyzoite-infected cells after 48 h of treatment with 240 µg/mL S-ATQ and EXO-ATQ compared to the untreated control group.

The effects of S-ATQ, J774A.1 EXO, and EXO-ATQ against *T. gondii* infection in Vero cells were determined. In this regard, the infection rate and the mean number of intracellular tachyzoites in the infected cells were examined by optical microscopy after 48 h of incubation. The results showed that S-ATQ and EXO-ATQ induced a dose-dependent decrease in the proliferation rate of *T. gondii*, RH strain tachyzoites. The results demonstrated that EXO-ATQ significantly reduced the infection rate of *T. gondii* tachyzoites compared to S-ATQ (*P*  ≤  0.05; Fig. 4).

### *In vivo* experiments

#### Toxicity studies

**Fig 5:**
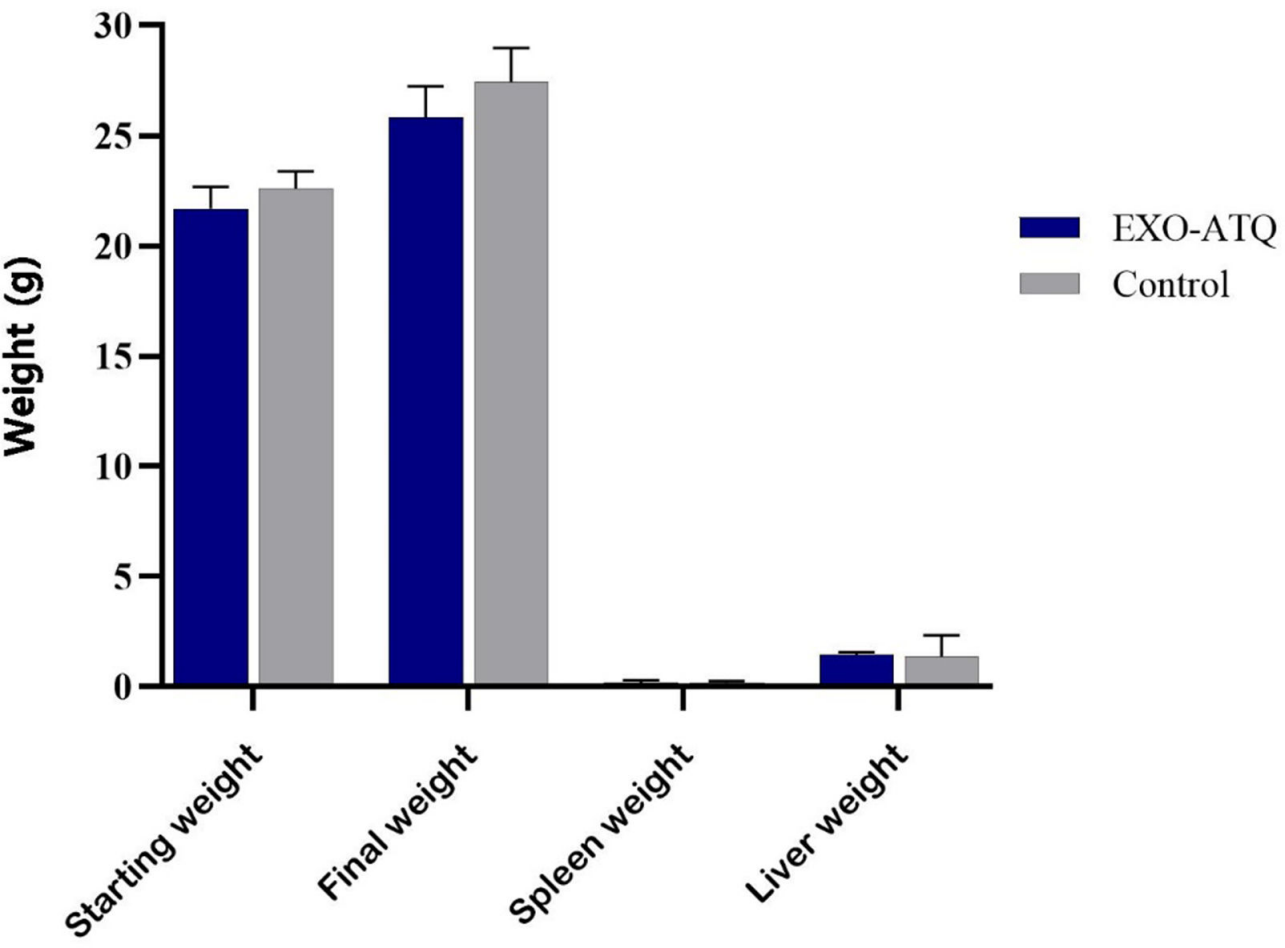
Toxicity studies including mice body, spleen, and liver weights in EXO-ATQ-treated and control groups. Results are presented as mean ± SD of four individual mice per group. No statistical differences were observed between the two groups (*P* > 0.05). Independent samples *t* test was used to make comparisons between groups.

**Fig 6:**
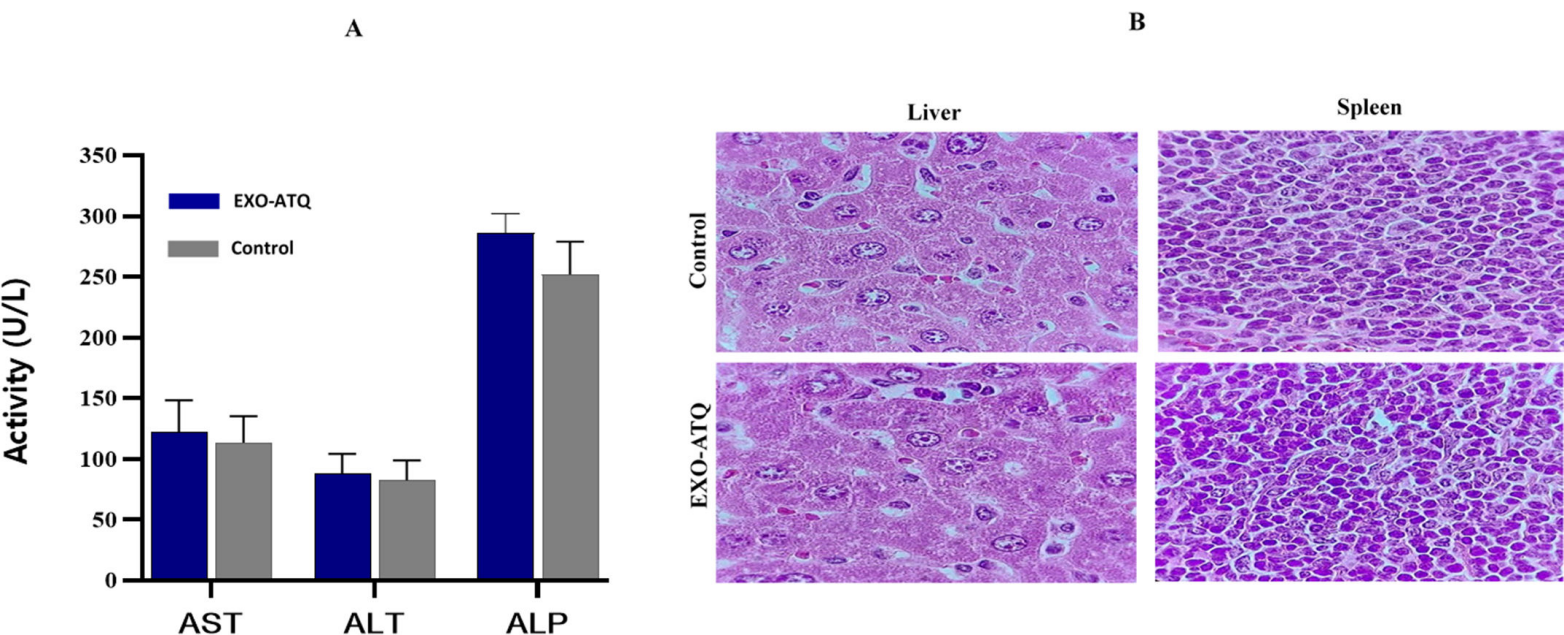
(A) Serum levels of AST, ALT, and ALP of mice in the control and EXO-ATQ treatment groups. Values are expressed as mean  ±  SD of the mean. No significant difference between groups was observed (*P* > 0.05). Independent samples *t* test was used to make comparisons between groups. (**B**) Representative histological image of the liver and spleen of control and treated mice (magnification 1,000×). No histopathological abnormalities were observed in the treated mice compared with the control mice.

Before assessing the *in vivo* efficacy of EXO-ATQ in *T. gondii*-infected mice, the IV administration of this formulation was done on naive BALB/c mice for tolerability and compared with the control group. All mice in the two groups (control and EXO-ATQ treated mice) survived throughout the experimental period. Neither behavioral nor physical changes were observed in all treated mice compared to the control group. No statistical differences in body weight were observed between the two groups (*P* > 0.05), and the increase in body weight was normal. In addition, the results revealed no significant differences in liver and spleen weights in both groups after 3 weeks (Fig. 5). The liver biochemical parameters were measured to investigate the toxicity of EXO-ATQ. As shown in Fig. 6A, the administration of EXO-ATQ had no significant effects on ALT, AST, and ALP levels compared with the control group. Moreover, no gross lesions were identified, and histopathological results showed no significant abnormalities or treatment-related changes in liver and spleen tissues examined from mice in the treatment group (Fig. 6B).

#### Acute experiments

**Fig 7:**
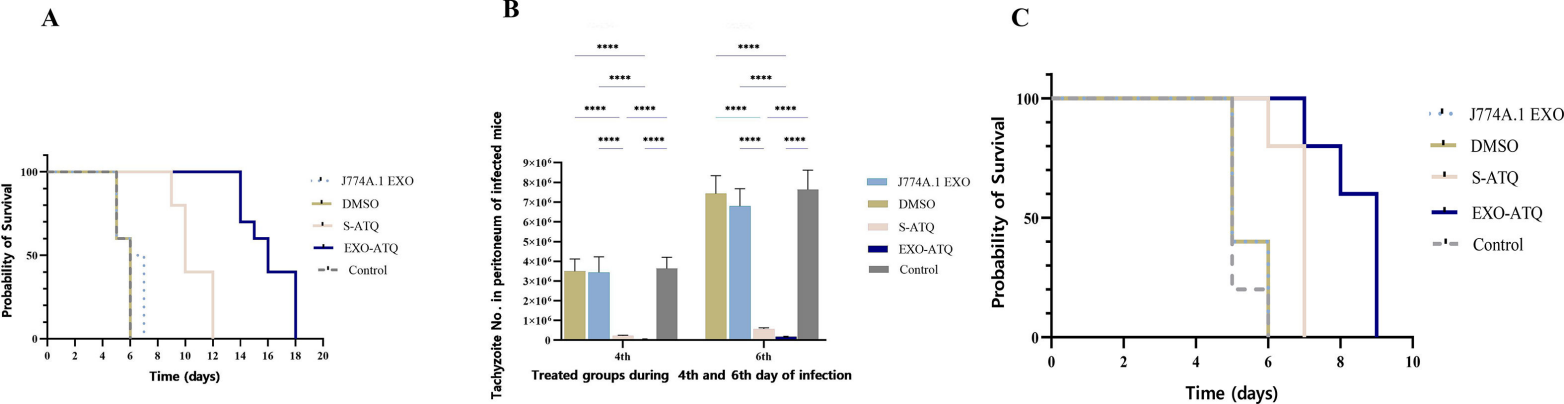
The anti-*Toxoplasma* efficiency of different compounds in female BALB/c mice infected with 1 × 10^4^ tachyzoites of *T. gondii*, RH strain, and virulence potential of post-treated tachyzoites. (**A**) The mice treated with EXO-ATQ had a significantly longer survival time than PBS-treated mice or those treated with S-ATQ, J774A.1 EXO, and DMSO. (**B**) The mean counts of peritoneum tachyzoites in mice treated with ATQ, especially EXO-ATQ, were significantly different from other groups. The data are presented as mean ± SD. ****P < 0.0001. One-way ANOVA was used to make comparisons. (**C**) Survival rates of the second group of mice inoculated with tachyzoites of *T. gondii* were obtained from the first group. The mice inoculated with tachyzoites obtained from EXO-ATQ-treated mice had a significantly longer survival time.

We evaluated the effects of EXO-ATQ in comparison with S-ATQ on RH strain of *T. gondii in vivo*. The mice survival time was assessed in treatment groups using the Kaplan–Meier method, followed by the log-rank test. As shown in the survival curve (Fig. 7A), compared to mice receiving J774A.1 EXO, DMSO, and PBS (all of these mice died within 7 days, *P* > 0.05), mice treated with S-ATQ and EXO-ATQ showed a prolonged survival time. Survival analyses revealed that the mice treated with EXO-ATQ had a considerably longer mean survival (ms) (ms = 16 days) than S-ATQ-treated mice (ms = 10 days; *P* < 0.005). Also, to investigate the anti-*T*. *gondii* effect of compounds, the number of tachyzoites in the peritoneal cavity was evaluated in mice. Results showed that treatment with S-ATQ or EXO-ATQ significantly inhibits the growth of tachyzoites in comparison with the J774A.1 EXO, DMSO, and PBS groups (Fig. 7B). The mean counts of peritoneum tachyzoites in mice treated with EXO-ATQ and S-ATQ were slightly different (*P* > 0.05). Growth inhibition rates of tachyzoites in mice receiving EXO-ATQ, S-ATQ, J774A.1 EXO, and DMSO on day 6 post-infection were 99.7%, 92.5%, 11%, and 2.8%, respectively, compared with those in the negative control group (Table 1). The virulence potential of post-treated tachyzoites was assessed by estimating the survival time of the second group that was inoculated with tachyzoites obtained from the first group. The results revealed prolongation of survival time in mice inoculated with tachyzoites obtained from S-ATQ- and EXO-ATQ-treated mice (Fig. 7C). The mean survival time of mice inoculated with S-ATQ- and EXO-ATQ-treated tachyzoites was 7 and 9 days, respectively, which was statistically significant (*P* < 0.05).

**TABLE 1:** Anti-*Toxoplasma* activities of EXO-ATQ, S-ATQ, J774A.1 EXO, and DMSO compared with control groups in the peritoneal cavity of mice infected with *T. gondii*, RH strain^*c*^

Group	No. of tachyzoites ± SD	% of growth inhibition^*a*^
J774A.1 EXO	6,804,000 ± 875,374	11
DMSO	7,432,000 ± 907,838	2.8
S-ATQ	574,000 ± 53,198^*b*^	92.5
EXO-ATQ	178,000 ± 23,875^*b*^	99.7
Control	7,646,000 ± 978,535	Reference^*d*^

^*a*^ Percentage of the growth inhibition = [(no. of tachyzoites in negative control − no. of tachyzoites after treatment)/no. of tachyzoites in negative control)] × 100.

^*b*^ Indication of statistically significant differences compared with the negative control (*P* < 0.0001).

^*c*^ Administration of S-ATQ or EXO-ATQ significantly inhibits parasite growth.

^*d*^ The control group served as a reference for estimating the tachyzoites reduction in the treated groups.

#### Chronic experiments

**Fig 8:**
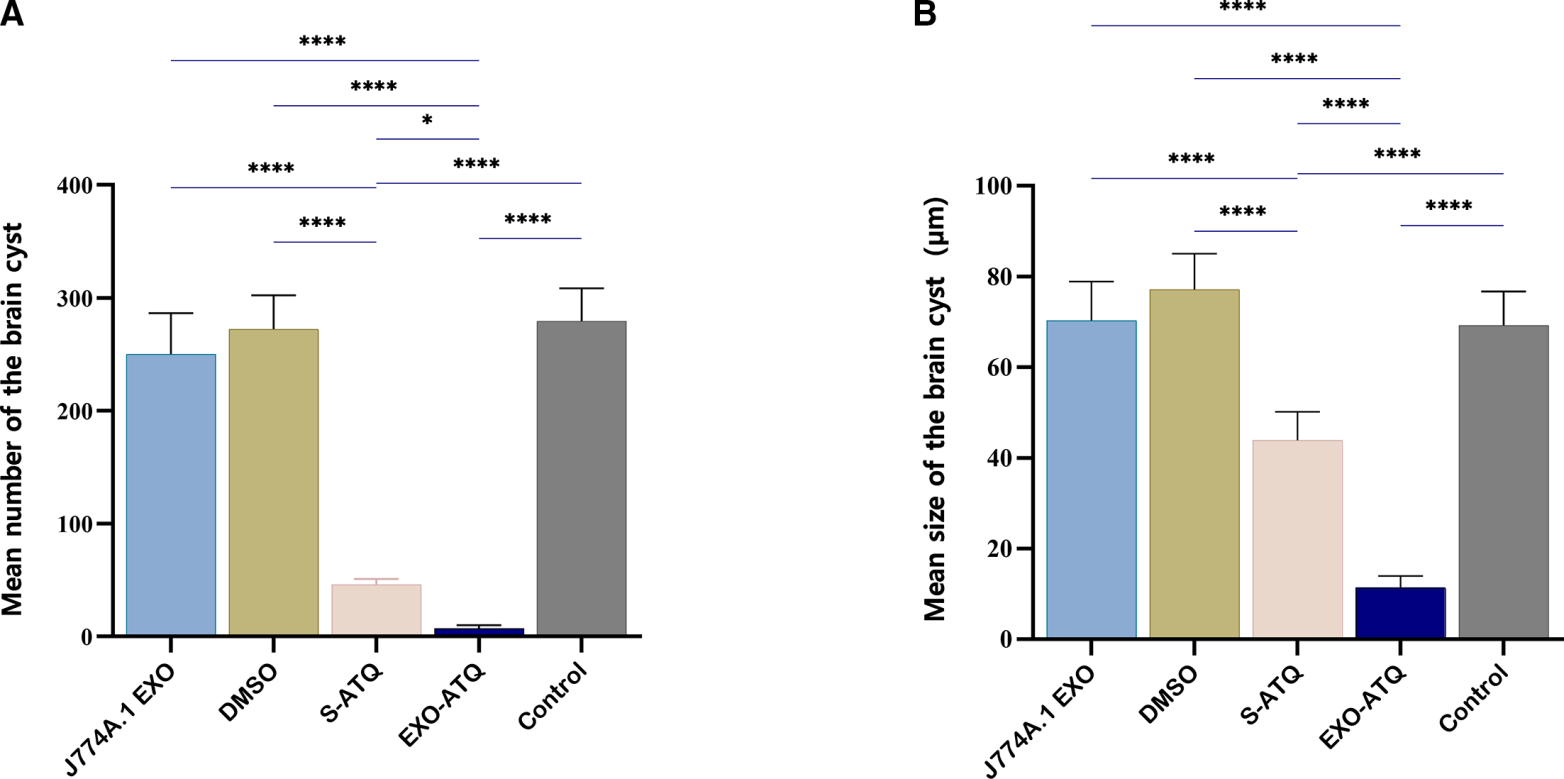
(A) The mean number and (**B**) size of tissue cysts in the brains of infected treated groups of mice compared to the infected non-treated control. The mean number and size of brain cysts were significantly reduced in the EXO-ATQ-treated group compared to the S-ATQ group. The data are presented as mean ± SD. One-way ANOVA was used to make comparisons between groups. *P < 0.05 and ****P < 0.0001.

The efficacy of EXO-ATQ was evaluated by comparing the number and size of brain tissue cysts of treated and control groups that were infected with *T. gondii*, Tehran strain. The mean number and size of brain tissue cysts in S-ATQ and EXO-ATQ-treated groups were significantly lower than other groups (*P* < 0.001; Fig. 8). The mean number and size of brain tissue cysts in EXO-ATQ-treated mice were 7.2 ± 2.8 and 11.3 ± 2.6 µm, respectively, and in S-ATQ-treated mice were 45.8 ± 5.1 and 43.8 ± 6.3 µm, respectively. The results indicated a statistically significant reduction in the mean number and size of cysts in the brains of EXO-ATQ-treated mice compared to the S-ATQ-treated group (*P* < 0.05). The results of %R of cysts are shown in Table 2.

**TABLE 2:** Effects of used compounds on brain cysts of infected treated groups of mice compared to the infected non-treated control

Group	No. of brain cysts ± SD	% of cyst reduction^*a*^
J774A.1 EXO	250 ± 36.3	10.49
DMSO	272.2 ± 30.1	2.54
S-ATQ	45.8 ± 5.1^*b*^	83.6
EXO-ATQ	7.2 ± 2.8^*b*^	97.35
Control	279.3 ± 29.2	Reference^*c*^

^*a*^ Percentage of cyst reduction = [(number of cysts in the control group − number of cysts in the experimental group)/(number of cysts in the control group)] × 100.

^*b*^ Indication of statistically significant differences compared to the negative control (*P* < 0.0001).

^*c*^ The control group served as a reference for estimating the cyst reduction in the treated groups.

**Fig 9:**
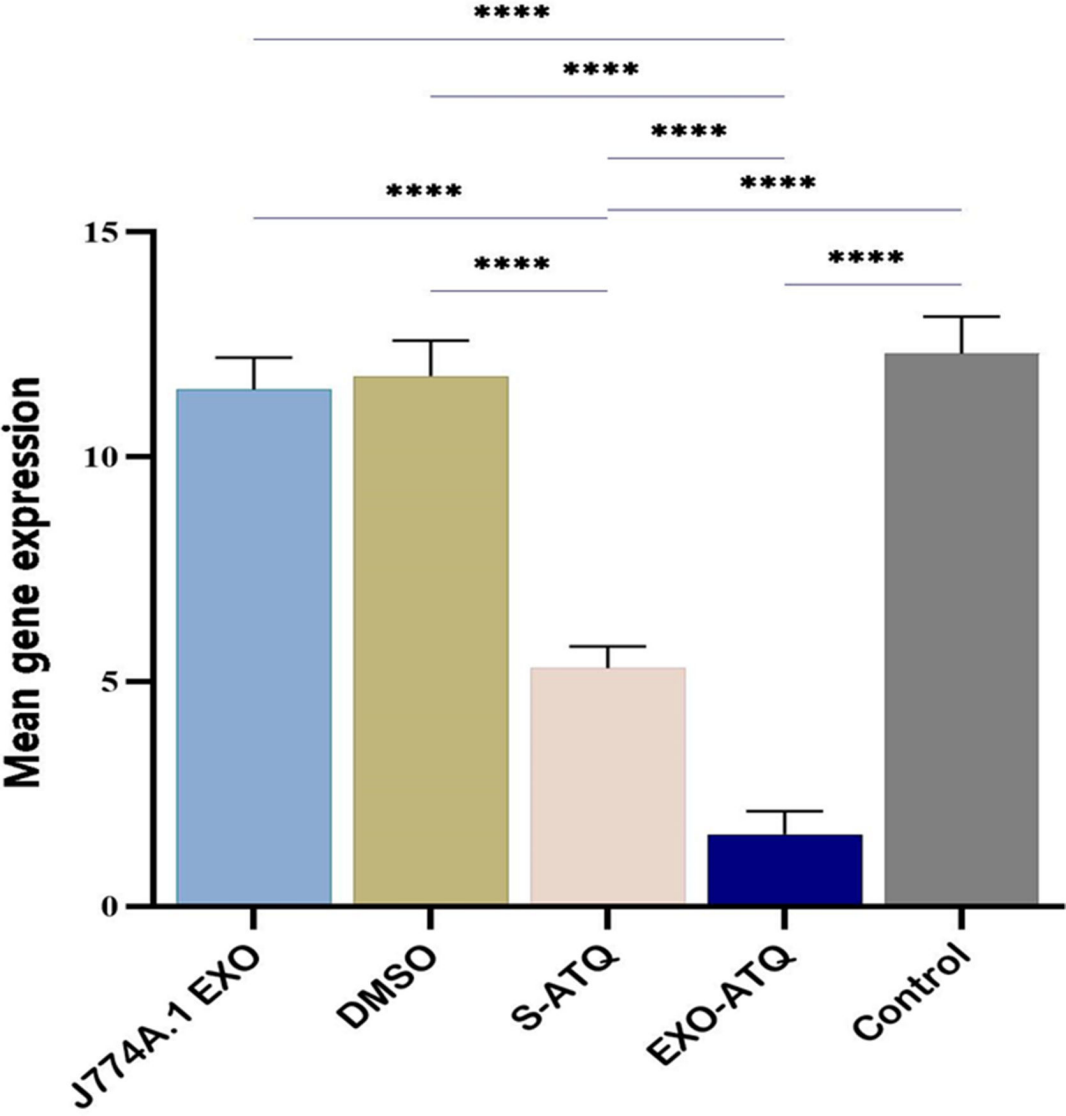
Mean expression ± SD of *BAG1* in infected treated groups of mice and infected non-treated control. The real-time PCR analysis represented a significant reduction of *BAG1* expression in the EXO-ATQ group compared to the other groups (*P* < 0.0001). One-way ANOVA was used to make comparisons between groups. ****P < 0.0001.

Real-time PCR for gene expression analysis was used to verify the effectiveness of EXO-ATQ in reducing brain tissue cysts of infected mice with *T. gondii*, Tehran strain. The results revealed no significant differences in the expression of BAG1 gene in J774A.1 EXO and DMSO-treated groups compared with that in the control group. However, down-regulation of BAG1 expression was seen in S-ATQ and EXO-ATQ treatment groups. Differences were statistically significant in the down-regulation of BAG1 expression in the EXO-ATQ group compared to the S-ATQ group (*P* < 0.001; Fig. 9).

**Fig 10:**
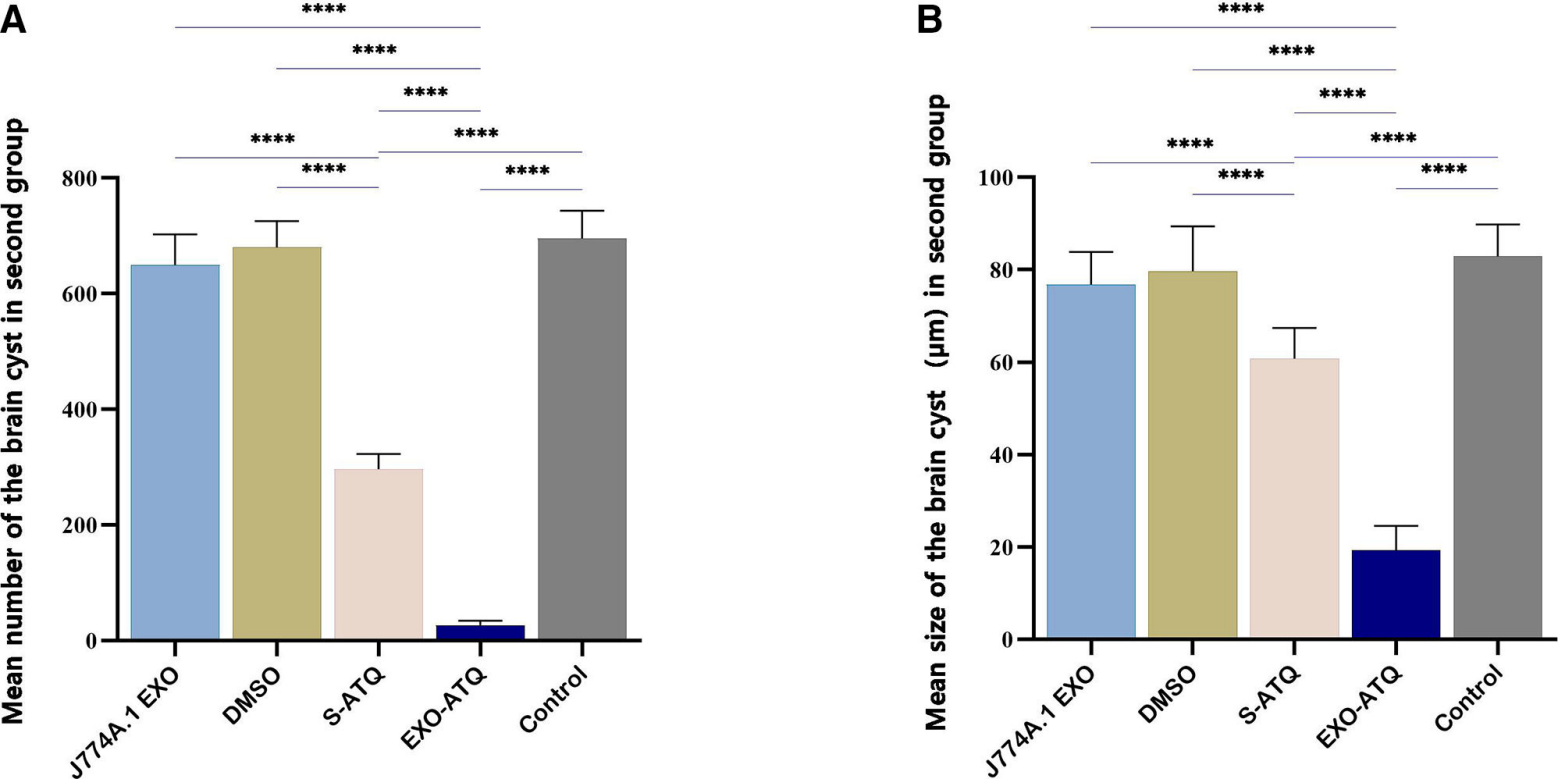
(A) The mean number and (**B**) size of tissue cysts in the brain of the second group of mice inoculated with bradyzoites obtained from the first group. Cyst burden in mice treated with EXO-ATQ was significantly different from those of S-ATQ-treated mice (*P* < 0.0001). One-way ANOVA was used to make comparisons between groups. ****P < 0.0001.

After the inoculation of brain suspensions from the first group of mice into new groups, a significant reduction in cyst burden was found in the inoculated mice with ATQ treated, especially EXO-ATQ brain suspension (*P*  ≤  0.0001). The mean number and size of brain tissue cysts in the second group of mice are shown in Fig. 10.

## DISCUSSION

Toxoplasmosis is an important disease that represents a global health threat (33). Albeit a variety of therapeutic regimens for toxoplasmosis have been developed, treatment of the chronic phase of this disease is still challenging due to poor drug delivery across the BBB (34). ATQ is an anti-toxoplasmosis drug that is known as the only successful treatment of the chronic phase, but it has poor efficacy due to its high hydrophobicity and low bioavailability (32). Nanotechnology and new formulations may find its way to improve the pharmacokinetic profile and efficacy of ATQ (35). A previous study showed that IV administration of ATQ nanosuspensions demonstrated a higher bioavailability and anti-*Toxoplasma* activity in murine model with reactivated toxoplasmosis compared with the suspension form of the drug (36). Moreover, IV administration of liposomal ATQ was 23 times more active than the free ATQ against *Leishmania infantum* in BALB/c mice model (37). In addition, ATQ nanoemulsion has been reported to be more effective than ATQ suspension against acute and chronic toxoplasmosis in a mouse model study by Jafarpour Azami et al. (32). Despite being widely used, artificial particles have many disadvantages such as toxicity and possible induction of immune response (20, 38). On the contrary, exosomes, as natural vehicles for drug delivery, can evade the reticuloendothelial system, so they are tolerated by the immune system (39). Furthermore, exosomes are stable in structure, size, and drug loading during circulation in the body (40). Clinical trials have confirmed the biocompatibility and safety of exosomes in humans and have introduced exosomes as good candidates for drug delivery. Although many studies have used exosomes as drug-delivery vehicles, no studies have been published on using exosomes as a drug-delivery system for treating toxoplasmosis. In the present study, for the first time, we determined the therapeutic potentials of EXO-ATQ against acute and chronic phases of *T. gondii in vitro* and *in vivo* conditions. The results of the present study proved the great potential of EXO-ATQ in the treatment of acute and chronic toxoplasmosis. In the present study, exosomes were isolated from macrophage as previous studies have recommended macrophage as a suitable source for exosomes due to their stability and ability to produce large amounts of these NPs. Moreover, macrophage exosomes can cross the BBB and deliver their cargo to the brain (17, 41, 42). The isolated exosomes were characterized based on morphology and size. According to the previously defined criteria (cup-shape morphology and the average size of 40 to 150 nm) (43), our results indicated that exosomes were successfully isolated from macrophage J774A.1. We also confirmed the identity of these particles as exosomes by investigating the presence of exosome-enriched proteins, CD9 and CD63, using bead-based flow cytometry assay. Our data showed the expression of CD9 and CD63 as determined in similar studies (44, 45). In order to load the exosomes with ATQ, the incubation method was used, and the loaded ATQ was quantified spectrophotometrically. Therapeutic agents can be loaded into exosomes with different methods, including electroporation, co-incubation, sonication, saponin treatment, freeze-thaw cycles, and extrusion (46). Co-incubation is a simple and usually efficient method for loading lipophilic drugs into exosomes (47), used to encapsulate ATQ (23), curcumin (48), and paclitaxel (49). Here, ATQ was loaded into exosomes by incubation for 12 h at room temperature under stirring, and the free ATQ was removed by passing through a 100 kDa amicon filter. The average loading efficiency of ATQ into exosomes was 57% ± 6% which can be due to the strong hydrophobic character of ATQ (log P: 5.2) that provided its efficient incorporation in the lipid bilayer of exosomes following incubation (23).

The results of evaluating the intracellular proliferation of *T. gondii* tachyzoites during the *in vitro* study showed the lowest percentage of infectivity in the EXO-ATQ group that was in agreement with a previous study done by Borgheti-Cardoso et al. They reported that ATQ-loaded pRBC exosomes were more efficient than equal amounts of free drug in decreasing parasite growth in an *in vitro Plasmodium falciparum* culture (23) which may be due to cellular membranes of exosomes and their extraordinary ability to interact with target cells (50). In addition, the exosomal surface is rich with integrins and tetraspanins that enable efficient attachment to the plasma membrane of target cells and release the cargo (51, 52).

*In vivo* toxicity of ATQ-loaded macrophage-derived exosomes indicated no behavioral and physical changes in all treated mice with EXO-ATQ. Moreover, histopathological results did not show any significant abnormalities in the liver and spleen tissues examined from mice in the treatment group. These results confirmed that exosomes could be applied as a safe drug delivery system (53).

In acute experimental toxoplasmosis, the mice treated with EXO-ATQ showed the longest mean survival time with a maximum of 18 days. Considerably, a prolonged survival rate was achieved in 100% of EXO-ATQ-treated mice at 10 days after tachyzoite inoculation, whereas 40% of mice survived in the S-ATQ-treated group, and no mice survived in the other groups, indicating that EXO-ATQ is more potent against acute toxoplasmosis.

In the context of parasite load, this study showed a statistically significant reduction in the tachyzoites count of the peritoneal exudates among S-ATQ and EXO-ATQ treated mice compared to that in other groups. Although S-ATQ showed a significant effect in the reduction of parasite load, its efficacy and potency were less than those of EXO-ATQ. These findings highlight the remarkable efficacy of EXO-ATQ against acute toxoplasmosis and elucidate that exosomes, as nanocarriers, exhibit long circulation time, good biocompatibility, endosome escape ability, and improve the interaction with target cells; thus, exosomes could enhance drug transport and therapeutic effects (54, 55). This result is consistent with other studies that showed that drug-loaded NPs could increase the survival time and reduce the parasite load in infected mice with *T. gondii*, RH strain (32, 56, 57). Hagras et al. used spiramycin-loaded NPs against acute experimental toxoplasmosis and compared its effect with free spiramycin. Their results revealed that spiramycin-loaded NPs induced a statistically significant increase in the mean survival time of mice with a substantial reduction in the mean tachyzoites count in the peritoneal cavity (56). In the study of Zawawy et al., the efficacy of triclosan (TS) and TS-loaded liposomes against the virulent strain of *T. gondii* was evaluated, and the results showed higher efficacy of liposomal encapsulation (57).

The most important finding of our investigation is that EXO-ATQ administration significantly decreased the mean number and average size of the brain cysts of chronically infected mice with *T. gondii*, Tehran strain. This reduction was about 97%, indicating a successful bypass of BBB. Notably, effective carriers can cross the BBB and deliver their cargo against brain tissue cysts in chronically infected mice with *T. gondii* (32, 35, 58). Jafarpour Azami et al. reported that treatment with nanoemulsion of ATQ eradicated tissue cysts in the brain of some infected mice completely (32). Another investigation showed that the use of SDS-coated ATQ nano-based suspensions promoted better therapeutic success in experimental toxoplasmosis models by improving the passage of BBB (35). In the study of Etewa et al. on chronically infected mice with *T. gondii*, a significant reduction in parasite count was observed in the spiramycin-loaded chitosan NPs treated group compared to the other subgroups (58).

To verify the microscopic studies, the relative expression of *BAG1* gene was evaluated using real-time RT-PCR. The results of reducing the number and size of tissue cysts in the microscopic examination were confirmed using the down-regulation of the *BAG1* gene. A similar observation was reported by Jafarpour et al., who used nanoemulsion of ATQ and curcumin in the treatment of experimental toxoplasmosis (32, 59).

Furthermore, we investigated the effects of every compound on the virulence potential of both RH and Tehran strains of *T. gondii*. For this purpose, tachyzoites and tissue cysts collected from treated and untreated groups of mice were inoculated into new groups. Then, animals were examined in terms of acute survival time and the number and size of brain tissue cysts in chronic infections. Results indicate that treatment with ATQ, especially EXO-ATQ, reduced the virulence potential of the parasite. In line with our results, it has been proven that the nanoemulsion of ATQ acts better than free ATQ in reducing the virulence potential of the parasite (32).

Despite several strengths associated with the present study, some limitations of this study should be considered: first, due to the presence of exosomes in FBS and inaccessibility to exosome-free FBS, to avoid their interference with macrophage exosomes, the mouse macrophage J774A.1 cells were adapted to the FBS-free medium through sequential adaptation. Although no significant morphology changes were detected after the adaptation process, it may affect the quality and quantity of exosomes produced. Second, although EXO-ATQ showed a significant decrease in the tachyzoite count of the peritoneal cavity of mice infected with RH strain and brain cysts of chronically infected mice, no complete parasite eradication was identified in our study. Finally, in the present study, we used untargeted exosomes for drug delivery, which limits the ability of exosomes to target the parasite. Targeted delivery can efficiently promote the local concentration of therapeutics without any adverse side effects.

In conclusion, this study confirmed that EXO-ATQ elicits potent anti-toxoplasmosis activity, which results in decreased parasite load and prolonged survival time in experimentally infected mice. This study also provided evidence that J774A.1 EXO could be used as a safe and suitable candidate for drug delivery; however, more studies are required to approve these findings, especially in clinical settings.

## Data Availability

The data sets generated and analyzed during the current study may be made available from the corresponding author on request.
